# A New Pain Scale

**DOI:** 10.1007/s11606-025-09593-5

**Published:** 2025-05-16

**Authors:** Laura M. Holzman

**Affiliations:** 1 Department of Art Education, Art History, and Art Therapy, Herron School of Art + Design, Indiana University, Indianapolis, IN USA; 2 Museum Studies Program, School of Liberal Arts, Indiana University, Indianapolis, USA

**Keywords:** art, pain, physical therapy

When I started the most recent round of physical therapy for my chronic pain, I braced myself for the inevitable. *On a scale from 1 to 10, how would you rate your pain today?*

That dreaded question. It was almost as bad as the deep muscle tightness that had stealthily brewed when I was pregnant and had become entrenched postpartum while my doctor ignored my reports of pain. The tightness that persisted for a year and a half before a physical therapist helped me start to release it. The tightness that crept back after two blissful years and landed me a referral for more physical therapy.

This was my first session with the new physical therapist, and I’d hoped to build a good relationship. So I took a deep breath and tried not to wince as the now-familiar thoughts flowed through my mind.

*How can I quantify such a qualitative experience*?

*It’s too complex to reduce to just one measure.*

*What’s really the difference between a 3 and a 4? Or a 6 and a 7?*

*I can imagine pretty extreme pain. This is way different from that. But if I report too low a number will you assume I don’t need to be here?*

*If I’d said something different to my old doctor, could I have avoided all of this?*

*What does it mean for pain to be worse? Is it about how long it’s lasted? How sharp or dull it feels? How annoyed or distracted or articulate or resilient I am on this particular day?*

*Is it a linear scale or a logarithmic scale? Or a little bit of both? This whole thing feels circular…*

I sighed and responded as politely and honestly as I could: “I know you have to ask me this, but I have a hard time with the pain scale. If you need me to tell you a number, I can make something up, but I don’t think it will be very meaningful.”

* * *

At each of our subsequent sessions, before we got to the business of identifying and releasing trigger points, stretching, and strength building, we first rehearsed the irritating pain scale exercise. I’d waffle and eventually conjure a number so we could move on. One week, we tried a version made for kids, where I picked from an array of facial expressions.

*But what if the smile is really a grimace?*

*And what if I’m crying on the inside but stoic on the outside?*

I liked the visual aspect of this tool, but it still felt inadequate and somewhat arbitrary. And I knew that the face was really just a placeholder; ultimately, whatever texture I thought was in there would be flattened into a number and entered into the patient management software.

* * *

One evening, as I did my prescribed exercises, the pain scale crept back into my mind and the numbers started to swirl. I couldn’t shake them, so I grabbed a pencil and started sketching. At my next appointment, I told my physical therapist that I’d designed a new pain scale. While she pressed her fingers into places I couldn’t reach on my own, I explained: “It still uses numbers, but it’s not about quantifying the pain. It’s more about the quality of the sensations. And,” I smiled, “it lets you be a 4 and a 7 at the same time.” She was intrigued, if also a little dubious. I told her I’d bring a copy to our next session.

* * *

**Figure 1:**
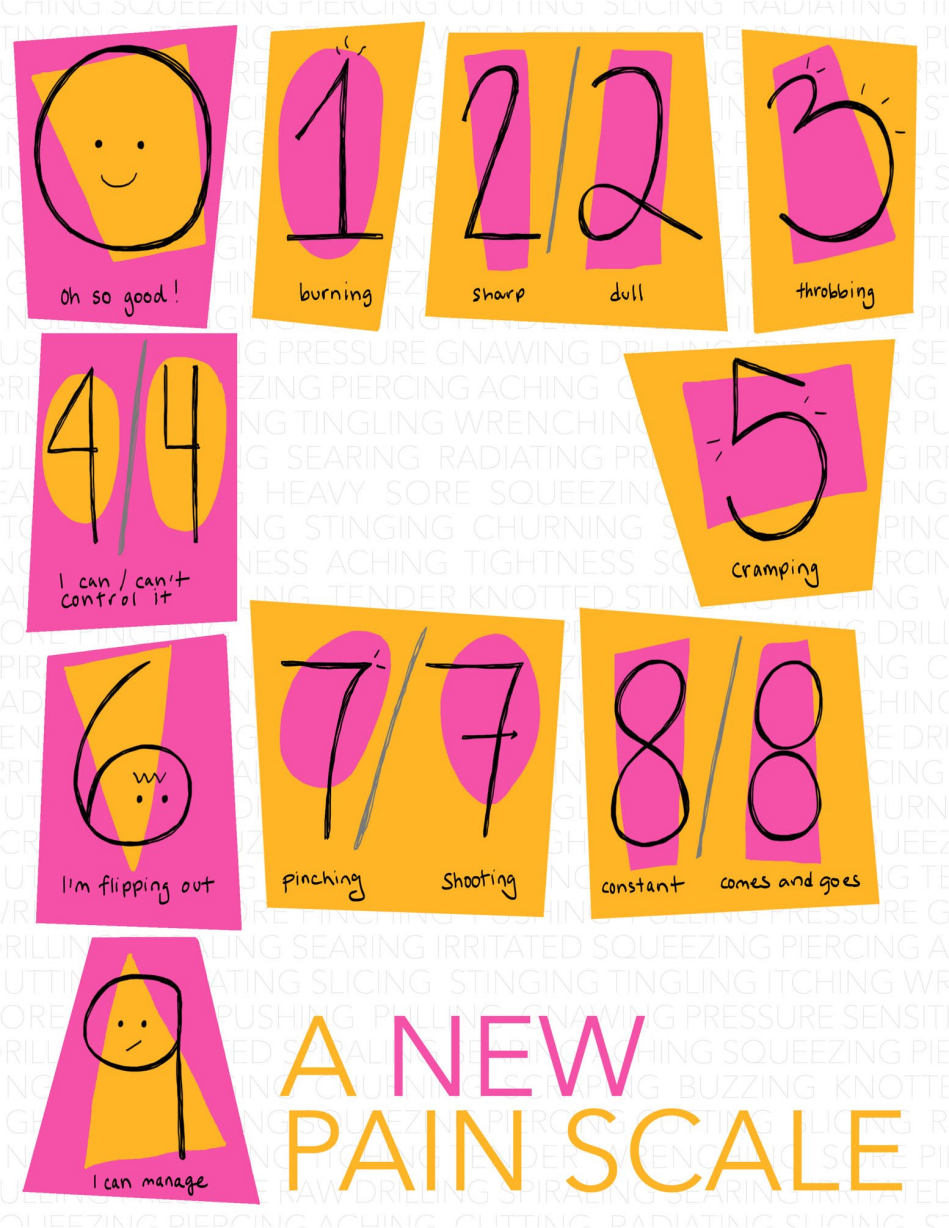
A New Pain Scale © 2024 by Laura M. Holzman.

Over time I’ve revised the design, but the key features have remained constant (Fig. 1). Handwritten elements ground it in human experience. Bright colors make it lively. The numerals are anchored in visual meaning — so much so that in some cases you can choose between different number forms to reflect how you’re feeling. If the pain is pinching, use an American-style seven. If the pain is shooting, write it the European way, with a line through the middle, like an arrow nocked on a bow. Pinching *and* shooting pain? Why not use both.

On a bad day, if I’m flipping out from a dull cramp that I can’t control, I would be a six, a looped two, a five, and an open four. On a better day, that same dull cramp might linger, but maybe I can control when it comes and goes, so I feel like I’m managing. I’d still be a looped two and a five, but I’d also be a closed four, an eight made from two stacked circles, and a nine.

What if the pain is stinging or searing? A blanket of other descriptors wraps around the background of the image. How could a single chart possibly encapsulate the full range of pain?

* * *

My physical therapist and I never used the new pain scale as a clinical tool. But imagining an alternative to the frustrating conventional measure allowed me to regain some of the individuality, complexity, and humor that the standard scale stripped from my healthcare experience. What if there were ways to describe, assess, and document pain that were more sensitive to the unique conditions of each patient? What if the process of reporting pain could even make a patient smile? My new pain scale might be more call to action than concrete solution, but patients like me would surely benefit from additional research into different ways of measuring and evaluating pain.

